# Challenges and future perspectives in using mesenchymal stem cells for efficient bone fracture healing

**DOI:** 10.3389/fbioe.2025.1568914

**Published:** 2025-05-30

**Authors:** Dong Han, Weiwei Liu, Jinpeng Gong, Yupeng Ma, Zhengwen Sun

**Affiliations:** Yantaishan Hospital, Trauma Orthopedics, Yantai, China

**Keywords:** osteogenesis, stem cell therapy, tissue regeneration, bone fracture, healing process

## Abstract

Mesenchymal stem cells (MSCs) demonstrate considerable potential for enhancing bone fracture healing due to their multipotency and immunomodulatory properties. This review investigates the relationship between MSCs, the immune system, and the skeletal microenvironment, focusing on the roles of cytokines and signaling pathways in osteogenesis. The healing process of bone fractures is complex and involves a coordinated response from various cell types, including immune cells and MSCs, which secrete bioactive molecules that promote tissue regeneration and modulate inflammation. Despite their promise, challenges such as variability in MSC sources, ethical considerations, regulatory restrictions, and obstacles in achieving effective delivery and retention at fracture sites restrict their clinical application. Recent advancements in MSC-based therapies, including innovative biomaterials, three-dimensional bioprinting, and gene editing technologies, aim to improve the therapeutic efficacy of MSCs. In addition, strategies to rejuvenate aged MSCs and enhance their regenerative capabilities are critical for addressing age-related fractures, as the functionality of MSCs declines with age. Understanding the mechanisms underlying MSC action, including their paracrine signaling and interaction with the bone microenvironment, is essential for optimizing their therapeutic use. Addressing existing limitations in MSC research and application provides a comprehensive perspective on the future of MSC therapies in bone repair. This review discusses the transformative potential of MSCs in regenerative medicine and orthopedics, highlighting the need for further research to unlock their full capabilities and improve clinical outcomes in patients with bone injuries.

## 1 Introduction

Bone homeostasis is maintained not only by the musculoskeletal system but also other biological systems. Both immune and skeletal systems contain other regulatory molecules such as cytokines and signaling molecules (Cai et al., 2024). Several diseases, including erosive arthritis, spondyloarthropathy, fibrodysplasia ossificans progressiva, and bone fracture healing are affected by inflammation and its consequences for bone resorption and deposition (Blauvelt and Chiricozzi, 2018; Zambrano-Zaragoza and Gutiérrez-Franco, 2021; Diolintzi et al., 2024). For this reason, it is evident that a robust interplay exists between the immune system and the skeletal system, influencing both physiological health and pathological conditions. Bone fracture repair is a process that is supervised by the immune system during the healing process, where immune cells enter the site of the hematoma and release cytokines, which leads to inflammation (Adejuyigbe et al., 2023). Due to the inflammation that precedes new bone tissue formation, the immune system is essential for fracture healing (Maruyama et al., 2020). Bone healing duration is prolonged in individuals on immunosuppressive medications (Kao et al., 2023), indicating a higher incidence of nonunion in HIV patients. Altogether, these findings indicate that the immune system holds great potential as a viable target for the therapy of bone fractures. Various immune cells, including macrophages, T cells, and B cells, migrate to the injury site and contribute to healing bone fracture (Burgan et al., 2025). Effector molecules like IL-6, TNF-α, and IL-17A also play a major role in regulating the healing process (Torres et al., 2023).

Osteogenesis involves a series of coordinated cellular and molecular events that facilitate the formation of new bone tissue. This process is essential for restoring skeletal integrity following injuries like fractures. The skeletal lineage consists of various cells responsible for bone maintenance and repair, including osteoblasts, osteocytes, and chondrocytes, functioning both during normal physiology and in response to injuries. The skeletal cell types primarily contribute to the development of bone and cartilage, while the cells responsible for bone resorption, called osteoclasts, originate from the hematopoietic lineage. Regular bone homeostasis is held by a delicate equilibrium between the actions of osteoblasts and osteoclasts (Kim et al., 2021). However, as individuals age, particularly postmenopausal women, the activity of osteoclasts exceeds that of osteoblasts, leading to heightened bone resorption and weakened bones overall (Fischer and Haffner-Luntzer, 2022). This process includes a sequence of highly synchronized cellular and molecular activities that facilitate the development of new bone tissue. Osteoblasts are critical in bone formation, as they produce and secrete the extracellular matrix, primarily composed of type I collagen. This matrix serves as a framework for the development of new bone, assisting in the process of mineralization while providing structural stability (Hasegawa et al., 2022). Maintaining bone homeostasis and facilitating efficient healing relies on the careful equilibrium between osteoblast activity and osteoclast-mediated bone resorption. The differentiation of mesenchymal stem cells osteoblasts is controlled by multiple signaling pathways, such as the Wnt/β-catenin and Bone Morphogenetic Protein (BMP) pathways, which demonstrates the complex structure of the regulatory mechanisms that control osteogenesis (Thomas and Jaganathan, 2022; Hu et al., 2024). The interaction between these cells and pathways ensures that bone repair imitates embryonic development, enabling the restoration of both structure and functionality in the damaged skeletal system (Arya et al., 2024).

Mesenchymal stem cells (MSCs) are a specific type of stromal cell characterized by their ability to expand *in vitro* and differentiate into various lineages. They can be sourced from multiple tissues, including umbilical cord, endometrial polyps, menstrual blood, bone marrow, and adipose tissue, making these sources practical for experimental and clinical applications due to the accessibility and quantity available. Despite their promise, many challenges remain in achieving standardized methods for the isolation, expansion, and characterization of MSCs, which can hinder the comparison of outcomes (García-Muñoz and Vives, 2021; Zhou et al., 2021). Current scientific efforts aim to address these challenges and explore the full potential of MSCs in medical applications, particularly in immunotherapy and regenerative medicine (Mei et al., 2024).

In orthopedics, MSCs demonstrate significant potential for bone and cartilage tissue engineering, providing innovative approaches that may surpass traditional methods such as joint replacement or filling (Deng L. et al., 2022; Malige et al., 2024; Raza and Hassan, 2024). This review aims to elucidate the role of MSCs in osteogenesis, detailing their differentiation pathways, paracrine signaling, and interactions with the bone microenvironment. It also highlights recent advancements in MSC-based therapies, including innovative biomaterials and delivery methods that enhance their efficacy. Furthermore, the review critically addresses challenges such as variability in MSC sources, ethical considerations, and regulatory obstacles, while identifying future research directions and potential clinical applications to optimize the use of MSCs in bone fracture healing.

## 2 Physiology of bone in homeostasis and fracture damage

Bone is a dynamic organ that is essential for support, protection, and mineral storage. It is composed of a composite matrix of organic and inorganic materials, primarily collagen fibers and hydroxyapatite crystals, which provide strength and rigidity. The physiology of bone is maintained through a delicate balance between bone formation and resorption, a process regulated by various cell types, including osteoblasts, osteoclasts, and osteocytes.

### 2.1 Bone homeostasis

At the cellular level, bone homeostasis is maintained primarily by the coordinated activities of osteoblasts, osteoclasts, and osteocytes, each performing distinct yet interconnected roles (Wu et al., 2024). Osteoblasts, derived from mesenchymal stem cells, are specialized cells responsible for synthesizing and secreting the organic components of the extracellular bone matrix, predominantly type I collagen (Huang et al., 2025). Following matrix deposition, osteoblasts facilitate mineralization by promoting the deposition of hydroxyapatite crystals, thereby imparting rigidity and structural strength to the newly formed bone.

On the other hand, osteoclasts, multinucleated giant cells originating from hematopoietic stem cell precursors, mediate the resorption of bone tissue (Kitazawa et al., 2025). These cells adhere tightly to the bone surface and create specialized microenvironments known as resorption lacunae. Within these localized zones, osteoclasts secrete acidic substances and proteolytic enzymes, such as cathepsin K, that dissolve mineralized matrix and degrade organic components, respectively (Chen R. et al., 2022). This resorptive activity serves to remodel bone architecture in response to mechanical demands and contributes significantly to systemic calcium and phosphate homeostasis by releasing these minerals into the circulation.

Osteocytes, the most abundant cell type within bone tissue, represent terminally differentiated osteoblasts that become encapsulated within the mineralized bone matrix during the process of bone formation (Marahleh et al., 2023). These cells occupy small cavities known as lacunae and extend extensive dendritic processes through narrow channels termed canaliculi, enabling communication with adjacent osteocytes and cells on the bone surface. Osteocytes function as mechanosensors, detecting mechanical loading and translating these mechanical stimuli into biochemical signals that regulate bone remodeling. Through intricate signaling networks, osteocytes modulate the activities of osteoblasts and osteoclasts (Zhao et al., 2025), thereby finely adjusting bone formation and resorption in response to mechanical stress and hormonal fluctuations.

The dynamic interplay between bone-forming and bone-resorbing cells is tightly regulated by various systemic and local factors, notably growth factors and cytokines, as discussed in section 3.5.

### 2.2 Bone damage and fractures

When bones are subjected to excessive mechanical stress or trauma, they can fracture, disrupting the normal physiological balance and integrity of the skeletal system. The healing process of fractures is a complex, multi-stage progression that ensures the restoration of bone structure and function (Molitoris et al., 2024). This complex process involves several overlapping stages, each playing a major role in the recovery and strengthening of the bone.

The initial stage of fracture healing is inflammation, which begins immediately after the injury occurs (Shu et al., 2024). At this point, a hematoma forms at the site of the fracture, serving as a foundation for the healing process. This hematoma is not merely a collection of blood but a dynamic environment that triggers an inflammatory response. Immune cells swiftly infiltrate the area, releasing a cascade of cytokines. These signaling molecules are vital as they promote healing and recruit MSCs to the site of injury. The presence of these cells sets the stage for subsequent phases of healing by laying the groundwork for tissue regeneration.

Following the inflammatory phase, the process transitions into the formation of a soft callus (Camarena et al., 2024). This phase typically occurs within a few days after the fracture. MSCs, now actively migrating to the fracture site, begin to differentiate into chondrocytes and osteoblasts. These cells are responsible for producing a fibrocartilaginous callus, which serves a critical function: stabilizing the fracture. This soft callus acts as a temporary scaffold, holding the bone fragments together and providing a framework for further healing.

As the healing process continues, the soft callus is gradually replaced by a hard callus in a phase known as hard callus formation (Trompet et al., 2024). This stage spans several weeks and marks the beginning of bone remodeling. Osteoblasts play a central role during this period, as they continue to secrete the extracellular matrix, which subsequently undergoes mineralization. The mineralized matrix forms a robust structure that significantly strengthens the fracture site, preparing it for the final phase of healing.

The concluding phase of fracture healing is bone remodeling, a process that can extend over months to years (Hente and Perren, 2021). During this time, the hard callus is meticulously remodeled into lamellar bone, thereby restoring the bone’s original structure and strength. This remodeling is a testament to the dynamic nature of bone, as it involves the coordinated activity of osteoblasts, osteoclasts, and MSCs. The interaction between these cells is essential for effective healing, ensuring that the bone regains its integrity and functionality.

### 2.3 Contribution of MSCs to bone homeostasis

MSCs are fundamental to maintaining tissue homeostasis, particularly in the context of bone health and repair. Their contributions are multifaceted and can be understood through various mechanisms that highlight their importance in skeletal integrity and function. In bone healing, MSCs are central through processes such as osteogenesis and chondrogenesis (Shen et al., 2024). During osteogenesis, MSCs differentiate into osteoblasts, facilitating the formation and mineralization of new bones, which is essential for restoring skeletal integrity after fractures. At the same time, in the early stages of fracture healing, MSCs can differentiate into chondrocytes, forming a cartilage scaffold that provides stability to the fracture site, setting the stage for further bone regeneration (Peng et al., 2023).

Beyond differentiation, MSCs engage in paracrine signaling, secreting a wide array of bioactive molecules, including growth factors, cytokines, and extracellular vesicles (Xue et al., 2023). This signaling plays a critical role in modulating the local microenvironment, thereby contributing to tissue homeostasis. A key aspect of this is the promotion of angiogenesis (Li et al., 2025). MSCs secrete factors like vascular endothelial growth factor, which enhance blood vessel formation, ensuring that the healing tissue receives adequate nutrients and oxygen. In addition, MSCs modulate inflammation by secreting anti-inflammatory cytokines, which help regulate the immune response, preventing excessive activation and ensuring a balanced healing process (Valencia et al., 2025). MSCs also engage in complex cellular interactions within the bone microenvironment, influencing the behavior and function of other cell types. For example, they can influence osteoclast activity by secreting factors that regulate RANKL and OPG levels, thereby modulating osteoclastogenesis and ensuring a balanced bone remodeling process. Moreover, MSCs provide support to endothelial cells, promoting vascular stability, which is essential for an efficient healing process.

Furthermore, MSCs are responsive to mechanical stimuli, a factor that is vital for maintaining bone density and health (Wang T. et al., 2025). Mechanical loading enhances MSC proliferation and their differentiation into osteoblasts, reinforcing the bone structure and facilitating adaptation to physical demands. This responsiveness to mechanical cues ensures that bones remain strong and capable of meeting the body’s functional needs.

Overall, MSCs are integral to maintaining bone homeostasis through their diverse roles in differentiation, signaling, interaction, and response to mechanical stimuli. Their ability to adapt and respond to various environmental factors highlights their significance in sustaining bone health and facilitating repair processes. The upcoming sections will explore the molecular mechanisms involved in more detail.

## 3 MSCs biology and mechanism of action

Several mechanisms of actions by MSCs contribute to their usefulness as a treatment option in regenerative medicine. They communicate using paracrine signaling as one of their primary functions (Xue et al., 2023). A variety of bioactive compounds including cytokines and growth factors are excreted by MSCs. The secretome plays a key role in modulating immune function and encouraging tissue regeneration (Zriek et al., 2021). MSCs produce essential molecules like vascular endothelial growth factor (VEGF) and transforming growth factor-beta (TGF-β) that are crucial for creating new blood vessels (angiogenesis) and healing damaged tissues (tissue regeneration) (Guillamat-Prats, 2021). MSCs can induce apoptosis in T lymphocytes during inflammation by activating the Fas-Fas ligand pathway which reduces inflammation (Vacaru et al., 2022). The connection between MSCs and their external environment controls their secretion activities and lets them alter their treatment results in relation to individual sicknesses. Their versatility is essential for maintaining tissue homeostasis and facilitating healing in inflammatory conditions, where they can modulate the inflammatory response.

**FIGURE 1:**
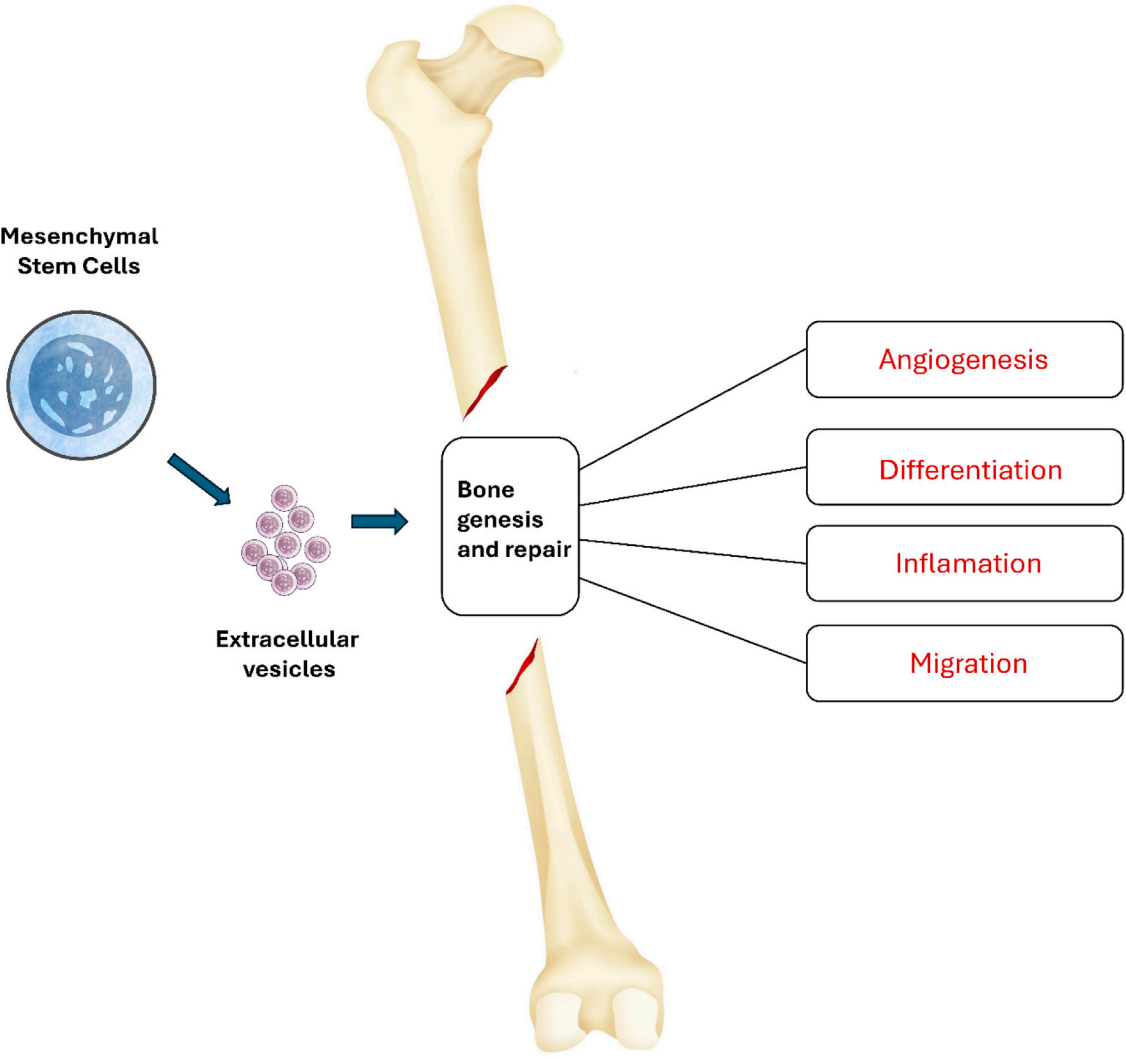
Mechanisms of action of MSC-EV in bone formation. MSC-EV can influence various processes associated with bone genesis and repair. These extracellular vehicles have the ability to enhance angiogenesis, reduce inflammation, and modulate migration and differentiation.

An important feature of MSCs involves their power to engage in cellular interactions and move biological elements to damaged cells (Matsuzaka and Yashiro, 2022). The survival and function of harmed cells are boosted by the action of tunneling nanotubes or microvesicles. In addition, the therapeutic advantages of MSCs are attributed to the significant role played by extracellular vesicles (EVs) produced by these cells (Tsiapalis and O'Driscoll, 2020; Mohd Noor and Abdullah, 2021; Sarhadi and Daddali, 2021). EVs transport mRNA, proteins, and microRNAs that have the potential to impact the actions of the cells they are received by. As presented in Figure 1, these extracellular vehicles have the ability to enhance angiogenesis, reduce inflammation, and modulate migration and differentiation, essentially imitating the actions of mesenchymal stem cells (Li F. et al., 2022).

The intricate nature of MSC processes, which encompass their ability to modulate the immune system, reduce inflammation, and promote tissue regeneration, highlights their promise as a therapeutic intervention in diverse clinical settings. Nevertheless, the specific pathways and interactions involved in this process are still being actively investigated, requiring additional understanding to enhance the effectiveness of MSC-based therapies for clinical applications (Wang et al., 2021).

### 3.1 Sources of mesenchymal stem cells

**FIGURE 2:**
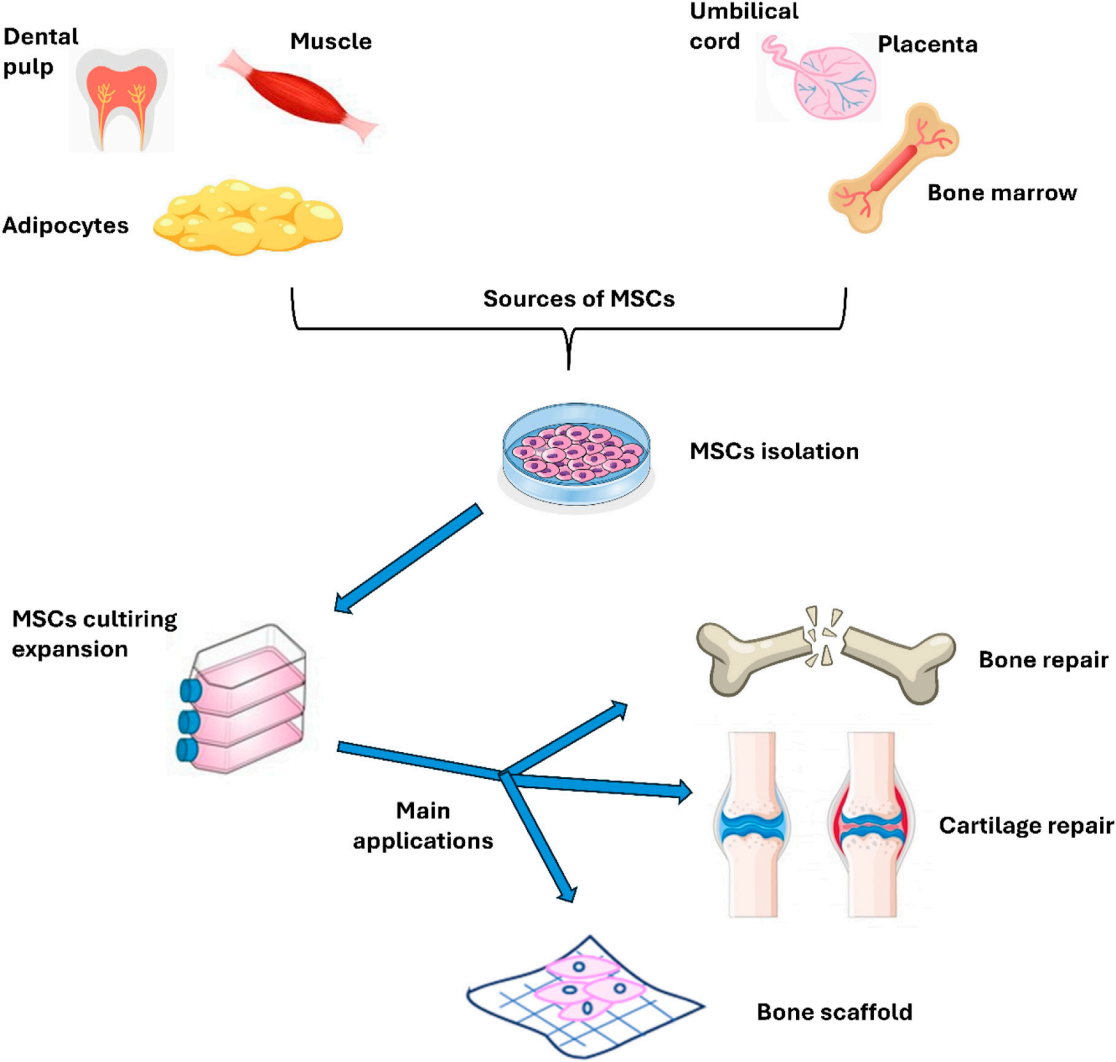
Sources and main uses of MSC in bone research.

MSCs are derived from different sources including bone marrow, adipose tissue, placenta, umbilical cord, and Wharton’s jelly. These versatile cells can be used in several ways, either by loading them within a scaffold or using them as a cell suspension for regenerative purposes, particularly in the context of efficient bone fracture healing. Figure 2 illustrates the sources and primary applications of MSCs in bone research, emphasizing their critical role in regenerative medicine and tissue engineering. The diverse origins of MSCs enhance their unique properties, making them especially valuable in promoting rapid and effective healing of bone fractures. In bone research, MSCs may be used to a variety of applications, including fracture healing (enhance the healing process of bone fractures by promoting osteogenesis and angiogenesis), bone defect repair (in the treatment of critical-sized bone defects, either alone or in combination with biomaterials), scaffold development (combined with biomaterials to create scaffolds for bone tissue engineering, aiming to replicate the natural bone environment) and 3D bioprinting (to create bone constructs for research and therapeutic applications), as discussed in the next sections.

#### 3.1.1 Bone marrow derived MSCs

The adult pluripotent stem cells sourced from bone marrow consist of endothelial progenitor cells, hematopoietic stem cells, and MSCs. Among all the tissues and organs, adipose tissue and bone marrow are most suitable because they are easily available and contain a higher number of precursor cells without any ethical concern. In comparing ADMSCs and BMMSCs, it is observed that, like ADMSCs, BMMSCs can modulate and differentiate into components of the immune system and hematopoietic tissue (Xu et al., 2023). BMMSCs play a protective role in maintaining pulmonary endothelial cell integrity by limiting endothelial barrier permeability, preserving adherent and tight junctions, and reducing inflammation. They have the ability to reduce both the permeability of blood vessels that results from hemorrhagic shock, and inflammation at the same time (Chen S. et al., 2022). These cells can also alleviate inflammation by influencing the activity of considerable cytokines such as TNF-α and IL-1 (Ahmed et al., 2021). The molecular changes induced by BMMSCs lead to increased oxygen tension in the affected tissue and improved lung function, while simultaneously reducing pro-inflammatory cytokines, lung tissue damage, and inflammation at the cellular level (Yang S. et al., 2022). Additionally, cytokines are released and lung regeneration is promoted while the inflammatory response to mechanical ventilation mediate lung injury is reduced (Yang Y. et al., 2022). Adult stem cells, specifically BMMSCs, exhibit several properties that make them suitable for stimulating bone repair. BMMSCs have the ability to transform into specific cells in tissues to restore their lost structure and function. They also release a variety of bioactive substances that contribute to the creation of a healing environment (Soliman and Ali, 2022). These substances have anti-apoptotic effects, regulate the immune system, and promote the growth of endothelial progenitor cells. A recent study indicates that allogeneic BMMSCs promote growth in children with osteogenesis imperfecta (Infante et al., 2021). In addition, BMMSCs were initially used for accelerating hematopoiesis due to their capacity to undergo differentiation into many cell types and release cytokines and growth factors. BMMSCs present immunomodulatory characteristics that enable them to repair injured tissue using paracrine and endocrine processes (Sid-Otmane et al., 2020). For these reasons, using allogenic nonimmunogenic BMMSCs would be a more viable approach from a clinical standpoint. The potential function of BMMSCs in facilitating the attachment of organs and preventing rejection may have multiple variables and could rely on the release of soluble growth factors, promoting the formation of new blood vessels, inhibiting immune cells that respond against foreign tissue.

#### 3.1.2 Adipose tissue-derived MSCs

Adipose tissue is distributed throughout various anatomical locations in the adult human body, including the bone marrow, synovial joints, subcutaneous layers, and internal organs. In addition, it may be present in ectopic sites such as the liver and skeletal muscles. This widespread presence of adipose tissue is significant, as it houses adipose-derived stem cells (ASCs), which have emerged as key modulators of tissue regeneration (Bunnell, 2021). These stem cells play a major role in the repair and regeneration processes by secreting specific soluble factors that facilitate cellular communication and promote healing (Krawczenko and Klimczak, 2022). ASCs release a variety of growth factors, including basic fibroblast growth factor, vascular endothelial growth factor, insulin-like growth factor 1 (IGF-1), and transforming growth factor-beta 1 (TGF-β1) (Liu et al., 2020).

When cultured in a specialized medium that promotes osteogenesis, enriched with factors such as bone morphogenetic protein-2 (BMP-2) and 1,25-dihydroxyvitamin D3, ASCs undergo a transformation into osteoblast-like cells over a period of 2–4 weeks (Skubis-Sikora and Sikora, 2023). Following this differentiation, these osteoblast-like cells initiate the synthesis of calcium phosphate within the extracellular matrix. This mineralization process can be assessed using staining techniques such as Alizarin Red or von Kossa, which facilitate the identification of osteocytes.

During the osteogenic process, several genes are upregulated, including alkaline phosphatase, type I collagen, BMP receptor 2, osteopontin, osteocalcin, bone sialoprotein, and BMP receptor 1 (Chan et al., 2021). In particular, male ASCs present a higher differentiation rate into osteogenic lineages compared to their female counterparts, and this differentiation is characterized by increased efficiency (Lee et al., 2021).

Rigotti and Marchi (2007) demonstrated the therapeutic potential of ASCs in ameliorating severe wound healing complications, including tissue atrophy, retraction, fibrosis, and radiation-induced damage. In their study, the authors explored how ASCs can promote healing by enhancing cellular regeneration and reducing inflammation in affected areas. They found that ASCs present unique properties that allow them to differentiate into various cell types, secrete growth factors, and modulate the immune response, all of which are of primary importance for effective wound healing. Furthermore, Rigotti et al. emphasized that the application of ASCs could significantly improve the healing process in patients suffering from chronic wounds or those undergoing treatments that compromise tissue integrity, such as radiation therapy. Using ASCs presents the potential to accelerate healing while simultaneously restoring the structural and functional integrity of damaged tissue. This dual benefit ultimately leads to improved patient outcomes and enhances quality of life.

#### 3.1.3 Other sources of MSCs

While bone marrow and adipose tissue have been the predominant sources for MSC isolation, a variety of other tissues have been identified as rich reservoirs of MSCs, each presenting unique biological characteristics and potential therapeutic applications.

The placenta is a promising source of MSCs, particularly from the chorionic villi and decidua. Placental MSCs (PMSCs) present a higher proliferation rate and enhanced immunomodulatory properties compared to BM-derived MSCs. These cells demonstrate a unique ability to modulate immune responses, which is particularly advantageous in the context of allogeneic transplantation and autoimmune disorders (Yang et al., 2021). The immunophenotypic profile of PMSCs, characterized by low expression of major histocompatibility complex class II and co-stimulatory molecules, further supports their potential in therapeutic applications aimed at minimizing immune rejection (Sarıkaya et al., 2022).

Umbilical cord-derived MSCs (UCMSCs) represent another promising source of MSCs, isolated primarily from Wharton’s jelly, a gelatinous substance that surrounds the umbilical vessels. UCMSCs present a distinct immunophenotype and exhibit robust multilineage differentiation potential, enabling them to differentiate into osteogenic, chondrogenic, and adipogenic lineages. Their naïve immunological status reduces the risk of graft-versus-host disease, making UCMSCs particularly appealing for regenerative therapies in various clinical contexts, including neurological disorders, cardiovascular diseases, and orthopedic injuries (Chen et al., 2024; Cui et al., 2022; Deng et al., 2024).

Dental pulp, the innermost soft tissue of teeth, has also been identified as a valuable source of MSCs. Dental pulp stem cells (DPSCs) can be harvested with minimal invasiveness, typically from extracted teeth, and possess significant multilineage differentiation capabilities. DPSCs have demonstrated potential in dental regeneration, pulp tissue engineering, and craniofacial reconstruction, with studies indicating their ability to differentiate into odontoblast-like cells, neurons, and other lineages (Lee et al., 2021; Bai L. et al., 2023). This accessibility and versatility position DPSCs as a critical resource in both regenerative dentistry and broader regenerative medicine applications. In addition, the efficacy of DPSCs combined with various scaffolds in enhancing bone regeneration in animal models have also been demonstrated (Namjoynik et al., 2023; Liu et al., 2024). Meanwhile, other studies have shown that pre-seeding scaffolds with DPSCs leads to improved bone healing, demonstrating their ability to support the regeneration of critical-size defects (Gendviliene et al., 2021). Furthermore, DPSCs present immunomodulatory properties, which can create a conducive microenvironment for healing by reducing inflammation (Imanishi et al., 2021). Their compatibility with different biomaterials further enhances their application, making DPSCs a promising alternative for treating bone defects and injuries (Alksne et al., 2022; Fujii et al., 2023). As ongoing research continues to explore their capabilities, DPSCs may significantly contribute to the development of innovative therapies for bone regeneration.

The synovial membrane, which lines the joints and is responsible for the production of synovial fluid, is another emerging source of MSCs. Synovial MSCs (SMSCs) exhibit unique properties, including the ability to produce hyaluronic acid and other synovial fluid components, which are essential for joint lubrication and homeostasis (Zamudio-Cuevas et al., 2022). SMSCs have shown promise in the treatment of osteoarthritis and other degenerative joint diseases, where their capacity to modulate inflammation and promote tissue repair can be harnessed to restore joint function (Sekiya et al., 2021; Furuoka et al., 2023).

In summary, the exploration of alternative sources of MSCs beyond bone marrow and adipose tissue is essential for advancing the field of regenerative medicine. Each of these sources (placenta, umbilical cord, dental pulp, and synovial membrane) provide distinct advantages, including enhanced differentiation potential, reduced immunogenicity, and accessibility. As research continues to elucidate the unique properties and therapeutic potentials of these MSC populations, the landscape of regenerative therapies will expand and contribute to novel treatment modalities aimed at a variety of orthopedic injuries and bone fracture healing.

Table 1 provides a comprehensive comparison of MSCs derived from the different sources previously discussed. The table analyzes key parameters such as proliferation rate, osteogenic differentiation efficiency, immunomodulatory capacity, harvesting complexity, and yield. Proliferation rate, measured in population doublings (PD), indicates the growth potential of MSCs. Osteogenic differentiation efficiency, assessed through alkaline phosphatase (ALP) activity and calcium nodule formation, reflects the ability of MSCs to differentiate into bone-forming cells. Immunomodulatory capacity is important for reducing inflammation and immune response and is measured by the ability of MSCs to suppress T cell activity. Harvesting complexity refers to the ease or difficulty associated with obtaining MSCs from each source, while yield indicates the quantity of MSCs that can be harvested.

**TABLE 1:** Comparative analysis of mesenchymal stem cells (MSCs) from various sources according to key parameters.

Source	Proliferation rate (PD times)	Osteogenic differentiation efficiency (ALP activity/Calcium nodule)	Immunomodulatory capacity (T Cell suppression rate)	Harvesting complexity	Yield
Bone Marrow	Moderate (20–30 PD)	High (ALP activity: 80–100 U/L)	High (70%–80% suppression)	High	Low
Adipose Tissue	High (30–40 PD)	Moderate (ALP activity: 60–80 U/L)	Moderate (60%–70% suppression)	Moderate	High
Umbilical Cord	Very High (40–50 PD)	Moderate to High (ALP activity: 70–90 U/L)	Very High (80%–90% suppression)	Low	High
Placenta	High (30–40 PD)	Moderate (ALP activity: 65–85 U/L)	High (75%–85% suppression)	Low	High
Dental Pulp	Moderate (20–30 PD)	Moderate (ALP activity: 60–80 U/L)	Moderate (60%–70% suppression)	Moderate	Low
Synovial Membrane	Moderate (25–35 PD)	High (ALP activity: 75–95 U/L)	High (70%–80% suppression)	Moderate	Moderate

The table highlights the diverse characteristics and practical considerations associated with MSCs from different sources. Each type of MSC provides unique advantages and limitations that influence their suitability for specific therapeutic applications. Bone marrow MSCs are known for their high osteogenic differentiation efficiency, making them ideal for bone regeneration therapies, though the invasive nature of harvesting and lower yield are significant drawbacks. Adipose tissue MSCs present a high proliferation rate and yield, with easier harvesting compared to bone marrow. Their moderate immunomodulatory capacity makes them suitable for a range of applications, although their osteogenic potential is slightly less than that of bone marrow MSCs. Umbilical cord MSCs exhibit very high proliferation rates and immunomodulatory capacity, with low harvesting complexity and high yield, making them promising candidates for regenerative therapies, particularly in immune modulation. Placenta MSCs share similar characteristics with umbilical cord MSCs, offering high proliferation rates and immunomodulatory capacity, along with easy harvesting and high yield, providing a viable option for large-scale therapeutic applications. Dental pulp MSCs present moderate proliferation and osteogenic differentiation, with relatively straightforward harvesting, making them noteworthy for potential use in dental and craniofacial regeneration, although their yield may be limited. Synovial membrane MSCs provide high osteogenic differentiation efficiency and immunomodulatory capacity, particularly relevant for therapies targeting joint and cartilage repair due to their origin and properties. Overall, the choice of MSC source should be guided by specific therapeutic goals, considering factors such as differentiation potential, immune modulation, ease of harvesting, and yield.

### 3.2 Characteristics and multipotency of MSCs

MSCs present two fundamental characteristics: the ability to differentiate into specific mature cell types (such as osteocytes, chondrocytes, myocytes, stromal cells of the bone marrow, tenocytes/ligamentocytes, adipocytes, dermal cells, and various connective tissue types) and the capacity to release a range of immunoregulatory bioactive macromolecules (Fernández-Francos et al., 2021). These molecules are essential for orchestrating restorative microenvironments within damaged tissues (Ehnert et al., 2021; Molitoris et al., 2024). MSCs are not exclusively localized in bone marrow; they can also be isolated from various tissues, including skeletal muscle, adipose tissue, cervical tissue, umbilical cord, amniotic fluid, and placenta. However, most of current research has primarily focused on MSCs derived from adult bone marrow, yielding substantial data on their biological characteristics and clinical implications. Often referred to as multipotent cells, MSCs are present in many adult tissues and exhibit self-renewal capabilities, potential for differentiation into diverse cell types, and rapid proliferation *in vitro*. When cultured in controlled environments, MSCs demonstrate three distinct biological properties that make them suitable for cellular therapy: (1) the ability to differentiate into multiple cell types, (2) the secretion of factors that facilitate tissue remodeling, and (3) the capacity to modulate immune responses.

The therapeutic efficacy of MSCs is attributed to their role as reservoirs of trophic factors (Fusi et al., 2023). Upon migrating to injured tissues to initiate repair, MSCs respond to local signals, including inflammatory cytokines, Toll-like receptor ligands, and hypoxic conditions. These stimuli can induce MSCs to synthesize the requisite growth factors essential for tissue repair and regeneration, some of which are implicated in angiogenesis and anti-apoptotic processes (Xu et al., 2023). Several clinical studies have reported the immunoregulatory functions of MSCs, which interact with dendritic cells, T lymphocytes, NK cells, and B lymphocytes (Razazian and Khosravi, 2021; Huang P. et al., 2022; Zhang et al., 2024). Immunoregulation by MSCs may occur *via* direct cell-to-cell contact or through the secretion of various factors. Consequently, MSCs can attenuate unwanted activation of T lymphocytes, promoting a tolerogenic state during tissue remodeling or repair, and inhibiting immune responses during the healing process. For this reason, MSCs are essential for maintaining immunological balance and influencing immune responses.

### 3.3 MSCs in the bone microenvironment

In tissue engineering and regenerative medicine, MSCs have shown promising results, especially in bone regeneration and the process of filling defects (Maheshwer et al., 2021). The use of MSCs in cell-based therapies provides multiple benefits. ASCs, in conjunction with BMMSCs, exhibit unique capabilities for differentiation and localization within bone tissue (Chen et al., 2024). This distinctive ability positions them as particularly effective candidates for enhancing bone regeneration. Their multipotent nature allows both ASCs and BM-MSCs to differentiate into osteogenic lineages, facilitating the repair and regeneration of bone structures. Furthermore, their inherent capacity to migrate to sites of injury and secrete bioactive factors contribute to an optimal microenvironment that supports bone healing processes.

**FIGURE 3:**
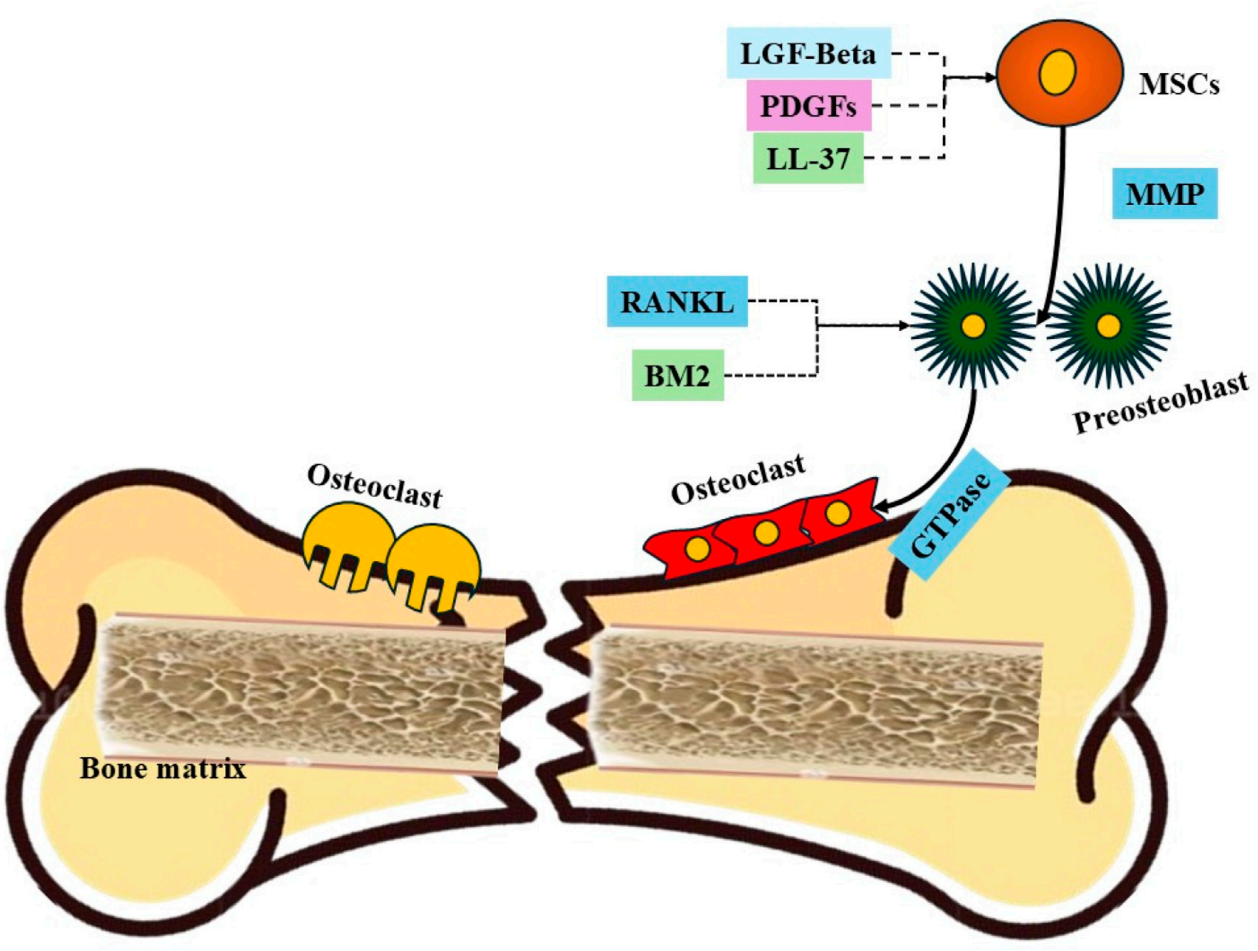
Proposed mechanism that MSCs migrate during the process of fracture healing. Key components include: (1) LGF-Beta and PDGF, which are released from the damaged bone matrix and platelets, respectively, serving as chemotactic agents that attract MSCs; (2) LL-37, an antimicrobial peptide that enhances inflammation and promotes MSC migration; (3) MMPs (Matrix Metalloproteinases), enzymes that degrade extracellular matrix components, facilitating the movement of MSCs; (4) Preosteoclasts activated by RANKL, promoting their differentiation into osteoclasts responsible for bone resorption; (5) GTPases, which regulate cytoskeletal dynamics and cell migration; and (6) the fractured bone and bone matrix, which provide the structural context for MSC migration and subsequent differentiation into osteoblasts and chondrocytes, essential for bone regeneration.

Figure 3 shows the proposed mechanism that MSCs migrate during the process of fracture healing. Initially, following a fracture, there is an inflammatory response that leads to the release of various growth factors and cytokines into the local environment. Key among these are Latent Growth Factor-beta (LGF-Beta) and Platelet-Derived Growth Factor (PDGF), which are released from the damaged bone matrix and platelets, respectively. These factors create a chemotactic gradient that attracts MSCs from the surrounding tissues to the fracture site. Concurrently, LL-37, an antimicrobial peptide, enhances the inflammatory response and further promotes MSC migration. As MSCs arrive at the injury site, Matrix Metalloproteinases (MMPs) facilitate their movement by degrading extracellular matrix components, thereby clearing a path for the migrating cells. Once at the fracture site, MSCs are influenced by the local microenvironment, including the presence of RANKL (Receptor Activator of Nuclear Factor Kappa-Β Ligand), which stimulates the differentiation of preosteoclasts into active osteoclasts. These osteoclasts are critical for bone resorption, allowing for the removal of damaged bone tissue and creating space for new bone formation. The MSCs, influenced by various signaling pathways, differentiate into osteoblasts and chondrocytes, contributing to the synthesis of new bone matrix. This complex interaction between MSCs, osteoclasts, and the signaling molecules ensures effective bone remodeling and restoration of structural integrity, leading to successful fracture healing.

Recent studies investigating the ability of human BM-MSCs and ASCs to generate bone tissue have shown inconsistent findings in terms of their osteogenic capability (Prajwal and Jeyaraman, 2022). There is ongoing debate about the comparative osteogenic capacity of these cell types; some studies suggest that BM-MSCs possess a superior capacity for osteogenesis, while others indicate that ASCs may have comparable or even enhanced potential (Midha et al., 2021; Kuca-Warnawin et al., 2023). Following a bone injury, the extracellular matrix of the bone releases a critical array of growth factors essential for the healing process. The bone remodeling cascade is orchestrated by several key signaling molecules, including VEGF, BMPs, and FGFs, among others (Sharma et al., 2021). Upon arrival at the injury site, MSCs can differentiate into osteoblasts, the primary cells responsible for bone tissue formation. This differentiation is initiated in response to inflammatory mediators, such as platelet-derived growth factors (PDGFs) and BMPs, which are secreted at the fracture site. These factors activate the BMP-Smad1/5/8 signaling pathway, promoting MSC migration to the damaged area (Huang et al., 2021). Additionally, in the context of injured bone, hypoxia-inducible factor 1-alpha (HIF-1α) enhances the production of stromal cell-derived factor-1 (SDF-1), further facilitating MSC migration to the injury site and thereby augmenting the bone regeneration process (Zhao et al., 2021).

### 3.4 Mechanisms of MSC-Mediated osteogenesis

The complex process of generating bones from precursors is governed by a dynamic interplay of signaling pathways, mechanical forces, and the biochemical environment surrounding MSCs. These factors collectively influence the differentiation of MSCs into osteoblasts, the cells responsible for bone formation. Central to the osteogenic differentiation of MSCs are several key signaling pathways, including the BMPs, Wnt, Hedgehog, and Notch pathways (Thomas and Jaganathan, 2022; Wu et al., 2024). BMPs are particularly relevant as they initiate and promote the osteogenic lineage commitment of MSCs. They activate downstream signaling cascades that enhance the expression of transcription factors such as Runx2 and Osterix (Pokrovskaya et al., 2020). These transcription factors are essential for the differentiation and functional performance of osteoblasts, driving the synthesis of bone matrix proteins and facilitating mineralization.

The Wnt signaling pathway also plays a significant role in MSC differentiation. Activation of Wnt signaling promotes the expression of osteogenic markers and inhibits the activity of osteoclasts, the cells responsible for bone resorption (Deng Z. et al., 2022). This dual action helps maintain a favorable environment for bone formation. Similarly, the Hedgehog and Notch pathways contribute to the regulation of MSC fate by modulating cell-cell interactions and influencing the balance between osteogenesis and adipogenesis (Wu et al., 2024).

In addition to these signaling pathways, the local microenvironment significantly impacts MSC behavior. Mechanical forces, such as those generated through physical activity or load-bearing exercises, can enhance bone formation by stimulating MSCs (Sun et al., 2022). Mechanical stimulation activates mechanotransduction pathways, which convert physical pressure into biochemical signals that promote osteogenic differentiation (Sun et al., 2022). This process highlights the importance of mechanical loading in bone health and regeneration, as it not only enhances MSC differentiation but also influences the overall architecture and density of the bone.

Novel studies have uncovered the involvement of MMPs in the differentiation of MSCs into osteocytes, a more mature form of osteoblasts embedded within the bone matrix. MMPs are enzymes responsible for the remodeling of the extracellular matrix, a critical process that supports the maturation and functionality of MSCs (Wu et al., 2020; Zhong-Sheng et al., 2022). By facilitating ECM remodeling, MMPs create a conducive environment for the proper differentiation of MSCs into functional osteocytes, thereby enhancing the overall bone regeneration process (Arai and Lee, 2023).

In summary, the mechanisms underlying MSC-mediated osteogenesis are complex and multifaceted, involving a combination of signaling pathways, mechanical forces, and biochemical signals. The interplay between these elements directs the way of MSCs toward osteoblasts and ensures the proper formation and maintenance of bone tissue. Understanding these mechanisms is essential for developing effective therapeutic strategies aimed at enhancing bone regeneration and treating conditions such as fractures and osteoporosis.

### 3.5 Growth factors and cytokines in MSC-mediated osteogenesis

In addition to the mechanisms previously discussed in section 3.4., a variety of cytokines and growth factors play essential roles in MSC signaling for osteoblast formation.

#### 3.5.1 BMPs (bone morphogenetic proteins)

BMPs are integral members of the TGF-β superfamily, playing a crucial role in the differentiation of MSCs into osteoblasts, which are essential for bone formation. Different BMPs have been increasingly recognized due to their therapeutic potential in treating bone disorders and facilitating fracture healing (Arai and Lee, 2023). These BMPs promote the recruitment of additional MSCs to sites of injury and also enhance the healing process by upregulating the expression of osteogenic markers and facilitating mineral deposition within the extracellular matrix (Wu et al., 2020; Zhong-Sheng et al., 2022).

Studies into BMP signaling pathways have elucidated their mechanisms of action in MSC differentiation. Upon binding to specific receptors on MSCs, BMPs activate downstream signaling cascades that stimulate the expression of key transcription factors such as Runx2 and Osterix. These transcription factors are pivotal for osteoblast differentiation and maturation, thereby driving the osteogenic program necessary for bone formation. Among the various BMPs, BMP9 has emerged as a particularly potent inducer of osteogenesis, demonstrating significant efficacy in both laboratory and clinical settings, particularly in applications such as spinal fusion and fracture repair (Mostafa et al., 2019; Liu et al., 2020). New evidence suggests that BMP9 may use distinct mechanistic pathways compared to other BMPs, providing insights that could be relevant for the development of personalized therapeutic strategies aimed at enhancing bone regeneration (Lu et al., 2023). The integration of BMPs with MSCs in tissue engineering approaches has shown promising results, significantly improving bone regeneration outcomes. This synergistic combination enables MSCs to not only generate bone tissue but also to promote the formation of biomaterials that closely mimic the characteristics of natural bone. Such biomimetic materials facilitate improved integration and functional recovery in clinical applications, highlighting the potential of BMP-MSC combinations in advancing regenerative medicine for bone repair.

#### 3.5.2 VEGF (vascular endothelial growth factor)

As MSCs and osteoblasts differentiate, they secrete VEGF, a key regulator in bone formation. In MSCs, VEGF significantly enhances the expression of osteogenic markers and promotes mineralization, acting as an intrinsic factor that supports osteoblast growth (Kim et al., 2021). Experimental data indicates that increased expression of VEGF-A in MSCs derived from trabecular bone correlates with heightened mineralization (Zha et al., 2021), highlighting its importance in the osteogenic process. On the contrary, disruptions in VEGF signaling decrease this effect.

VEGF regulates MSC osteogenesis through multiple signaling pathways. The development of RUNX2, a transcription factor for osteoblast differentiation, relies on the ERK signaling pathway. Additionally, VEGF plays a major role in maintaining the balance between adipogenesis and osteogenesis within the bone marrow (Burger et al., 2022). It promotes the production of osteoblasts while inhibiting the differentiation of MSCs into adipocytes. This regulatory function is particularly important in aging and osteoporosis, where the equilibrium between bone and fat cell formation is often disrupted.

In MSC-based therapies for bone restoration, the incorporation of VEGF demonstrates clear advantages. Studies into the combination of MSC therapy and VEGF aim to enhance treatment outcomes in challenging scenarios, such as non-healing fractures and osteoporosis-related bone degeneration (Lu et al., 2023). VEGF not only stimulates the growth of blood vessels but also supports bone formation, creating a favorable microenvironment for regeneration (Burger et al., 2022). A comprehensive understanding of the intricate pathways and mechanisms through which VEGF operates can lead to the development of targeted therapies. These advancements may enhance the capability of MSCs to promote bone development and improve bone regeneration, offering promising strategies for addressing various bone-related conditions.

#### 3.5.3 Cytokines

Cytokines are essential mediators in the process of MSC-mediated osteogenesis, significantly influencing the differentiation and functionality of mesenchymal stem cells in bone formation (Zhou et al., 2022; Hsu et al., 2023). Among the key cytokines involved, interleukin-6 (IL-6), interleukin-10 (IL-10), and tumor necrosis factor-alpha (TNF-α) are key factors in orchestrating the complex signaling networks that drive osteogenic processes.

IL-6 is known for its dual role in bone metabolism (Wang et al., 2021). It can promote osteoblast differentiation while also influencing the balance between bone formation and resorption. In the context of MSCs, IL-6 facilitates the transition of these stem cells into osteoblasts by enhancing the expression of osteogenic markers (Yang S. et al., 2022). This cytokine stimulates the proliferation of MSCs and also facilitates the recruitment of additional cells to the site of bone formation, thereby supporting the overall osteogenic process (Huang Y. et al., 2022).

On the other hand, IL-10 is primarily recognized for its anti-inflammatory properties, which are important in maintaining a favorable environment for bone regeneration. By suppressing excessive inflammatory responses, IL-10 helps to create a conducive microenvironment for MSC differentiation into osteoblasts. This cytokine enhances the survival and function of MSCs during the osteogenic process, promoting tissue repair and regeneration (Ni et al., 2022). The secretion of IL-10 by MSCs can also mitigate the negative effects of pro-inflammatory cytokines, thus ensuring that the osteogenic potential of MSCs is not compromised during inflammation (Yuan et al., 2021).

TNF-α, while often associated with promoting inflammation, also plays a complex role in osteogenesis. At moderate levels, TNF-α can stimulate MSCs to differentiate into osteoblasts, contributing to bone formation (Cao et al., 2021). However, excessive levels of TNF-α can lead to increased osteoclast activity, resulting in bone resorption (Yao et al., 2024). Therefore, the precise regulation of TNF-α levels is of primary relevance; MSCs can modulate its activity through the secretion of other cytokines, balancing the effects of TNF-α to favor bone formation over resorption.

The complex interaction of these cytokines in MSC-mediated osteogenesis highlights the significance of the local microenvironment in regulating stem cell behavior and bone regeneration (Wang et al., 2020). Understanding how IL-6, IL-10, and TNF-α influence MSC differentiation and function enables the development of targeted therapeutic strategies that enhance the regenerative potential of MSCs. This knowledge is particularly valuable for advancing treatments for bone-related conditions, as manipulating cytokine signaling could optimize the use of MSCs in regenerative medicine, ultimately leading to improved outcomes in bone healing and repair.

## 4 Functional enhancement of MSCs for osteogenesis

### 4.1 Scaffold-based approaches for MSC delivery and functionality

#### 4.1.1 Types of scaffolds

Bioscaffolds are essential components in bone tissue engineering, functioning synergistically with growth factors and stem cells to facilitate bone regeneration (Ravoor et al., 2021). These scaffolds act as reservoirs for various biological components and must meet specific criteria to be effective. An ideal scaffold should exhibit biocompatibility, ensuring no harmful effects on local or systemic tissues. It should promote regular cellular functions and facilitate critical processes such as cell attachment, proliferation, extracellular matrix deposition, and osteogenesis. In addition, the scaffold must stimulate angiogenesis, promoting blood vessel formation within weeks of implantation, while possessing suitable mechanical properties, optimal pore size, and an appropriate biodegradation rate to align with physiological conditions.

Calcium phosphate ceramics, which include hydroxyapatite (HA) and β-tricalcium phosphate (β-TCP), are widely used as synthetic scaffolds due to their excellent biocompatibility, high bioactivity, and osteoconductivity (Hoveidaei et al., 2023). The incorporation of β-TCP as a matrix along with varying concentrations of HA nanofibers into composite porous scaffolds significantly enhances their mechanical properties (Mohd Zaffarin and Ng, 2021). A β-TCP scaffold containing 5% HA nanofibers exhibited a compressive strength of 9.8 ± 0.3 MPa, comparable to the compressive strength range (2–10 MPa) of human cancellous bone. Furthermore, metallic materials such as stainless steel and cobalt-titanium alloys are commonly used in clinical orthopedics due to their favorable mechanical properties and biocompatibility. However, the lack of bio-specific recognition epitopes on the surfaces of these metallic scaffolds can diminish their biological activity. To enhance cellular interactions and improve the efficacy of tissue repair and regeneration, growth factors and other bioactive substances are often applied as surface coatings on these scaffolds.

In addition to ceramics and metals, both natural and synthetic polymer-based scaffolds exist, such as collagen and polycaprolactone. These polymers provide customizable porosity, allowing for tailored nutrient and cell infiltration for effective tissue integration and regeneration (Murab et al., 2023). Their mechanical properties can be engineered to match the specific requirements of bone tissue, providing necessary support while accommodating physiological loads. Additionally, the degradation profiles of these polymers can be fine-tuned to align with the rate of new tissue formation, ensuring that the scaffold maintains structural integrity long enough to support cellular activities before gradually being replaced by natural tissue (Wang et al., 2020; Kirmanidou et al., 2024). Many studies have investigated a range of polymeric materials used in bone healing applications, emphasizing their design considerations, fabrication techniques, and modifications aimed at enhancing scaffold performance (Ye et al., 2020; Collins et al., 2021; Dec et al., 2022; Koushik et al., 2023). This exploration highlights the potential of polymer scaffolds to mimic the natural extracellular matrix and facilitate cellular behavior, ultimately contributing to improved outcomes in bone regeneration therapies.

#### 4.1.2 Integration of MSCs with biomaterial scaffolds

Biomaterial scaffolds represent an advanced three-dimensional framework that closely mimics the natural extracellular matrix, which facilitates the adhesion, proliferation, and differentiation of MSCs. Scaffolds composed of bioceramics, particularly β-tricalcium phosphate (β-TCP), in conjunction with biodegradable polymers, significantly enhance the process of bone regeneration (Aoki et al., 2024). Various combinations of β-TCP and MSCs have shown efficacy in repairing large-scale bone defects in non-human primate models, suggesting their potential applicability in clinical settings for human patients (Cao et al., 2021; Zhou et al., 2021). These scaffolds not only meet the requisite mechanical properties for effective bone regeneration but also provide biochemical signals that promote the differentiation of MSCs into osteogenic lineages.

The interaction between MSCs and scaffolds involves multiple mechanisms, as MSCs secrete bioactive molecules in response to their immediate microenvironment, thereby promoting angiogenesis and modulating the immune response (Sheehy et al., 2021). Comparative evaluations indicate that the physical and chemical properties of the scaffold surface significantly influence MSC responses, including adhesion and differentiation (Bernardo et al., 2022). To enhance cellular activation on scaffold surfaces, various surface modification techniques have been used, leading to improved tissue compatibility and healing outcomes. For example, the incorporation of nano topographical features onto scaffolds enhances osteogenic differentiation by providing additional signals that guide MSC behavior (Xiao et al., 2024). This multifaceted approach demonstrates the importance of scaffold design in optimizing bone tissue engineering strategies.

### 4.2 Preconditioning and priming of MSCs

#### 4.2.1 Hypoxia preconditioning

This approach involves isolating MSCs and subjecting them to hypoxic conditions, which are characteristic of most injured tissues. Hypoxia preconditioning offers several advantages, significantly enhancing the survival, proliferation, migration, and differentiation capabilities of MSCs. Low oxygen levels, typically between 1% and 5%, optimize MSC viability and proliferation (Yang Y. et al., 2022). This improvement is primarily attributed to the activation of specific “wake-up” pathways, notably the hypoxia-inducible factor pathway, which regulates genes associated with cellular survival and metabolism. Consequently, hypoxia preconditioning may elevate the expression of pro-survival proteins and growth factors, thereby enhancing the regenerative potential of MSCs.

In addition to bolstering MSC survival, hypoxic conditions promote the paracrine properties of these cells. Hypoxia-conditioned MSCs (HCM-preconditioned) secrete an enriched secretome that includes growth factors, cytokines, and extracellular vesicles, which collectively exert angiogenic, anti-inflammatory, and tissue remodeling effects (Yang et al., 2023). This enhanced secretome contributes to the therapeutic efficacy of MSCs in various contexts, including ischemic injuries and inflammatory disorders. Furthermore, hypoxic conditions influence the multilineage differentiation potential of MSCs with recent studies demonstrating that hypoxia stimulates their differentiation into cartilage and bone tissue (Ma, 2024). As such, hypoxia-preconditioned MSCs hold significant promise for applications in bone and cartilage regeneration, particularly in the context of wound healing conditions such as fractures and osteoarthritis.

#### 4.2.2 Mechanical stimulation

Mechanical stimulation is essential for activating various signaling pathways in MSCs, with the RhoA/ROCK pathway being particularly significant. This pathway regulates the cytoskeletal composition and mediates cellular responses to mechanical stress (Sun et al., 2022). Activation of the RhoA/ROCK pathway facilitates the differentiation of MSCs into specific lineages, such as osteoblasts and chondrocytes (Saidova and Vorobjev, 2020), thereby enhancing their ability to generate bone and cartilage under appropriate mechanical conditions.

Mechanical stimulation can significantly enhance MSC proliferation, migration, and differentiation. Applying dynamic compression and tensile forces promotes the development of chondrogenic cells from MSCs, leading to increased cartilage production (Raman et al., 2022). This is especially relevant for restoring articular cartilage, as mechanical stimuli closely replicate the normal loading conditions experienced *in vivo*.

Additionally, mechanical stimulation is essential in bone regenerative therapies, enhancing MSC-mediated osteogenesis. Applying mechanical forces encourages MSCs to adopt specialized behaviors characteristic of osteoblasts, resulting in improved bone formation and healing, particularly in fracture models (Sun et al., 2022). This approach is beneficial for non-union fractures, where mechanical signals can elicit the necessary biological responses to facilitate successful healing. Integrating mechanical stimulation into MSC therapies presents a promising strategy for enhancing tissue regeneration and repair.

## 5 Current trends in MSC-Based therapies for bone fracture healing

Advances in MSC-based treatments for bone fracture healing have emerged as a transformative approach in regenerative medicine, particularly in addressing hereditary, traumatic, or degenerative conditions affecting bone, joints, and cartilage. The potential clinical applications for MSCs continue to expand, especially in the context of treating non-unions fracture, where traditional healing methods often fail. Empirical studies indicate that the application of MSCs, either independently or in conjunction with biodegradable scaffolds, significantly enhances the healing process in patients with non-unions (Khatkar and See, 2021). Preliminary clinical studies have shown that patients receiving autologous BMSCs present considerable improvements in the Tomographic Union Score and a reduction in pain at the fracture site (Jayankura et al., 2021). This approach demonstrates the potential of using a patient’s own MSCs to facilitate healing while minimizing the risk of immunological rejection. Furthermore, studies exploring the effects of BMSCs in individuals with osteoarthritis indicate promising results. A phase I/IIa clinical trial conducted by Chahal et al. (2019) involving participants with advanced Kellgren–Lawrence knee osteoarthritis demonstrated significant improvements in post-surgery Knee Injury and Osteoarthritis Outcome Scores related to pain, symptoms, and quality of life, particularly with higher doses correlating to reduced levels of cartilage catabolic biomarkers and MRI-detected synovitis.

In addition, the ability of MSCs to migrate to sites of inflammatory diseases allows them to exert localized effects on inflammatory and immune-mediated tissue damage, promoting tissue recovery. Studies on primates have illustrated that when BMSCs are administered intravenously, they disperse across various tissues, indicating their therapeutic potential (Devine et al., 2023). Among the different types of MSCs, BMSCs are recognized as the most advanced in terms of therapeutic application, which highlights their significant promise in clinical settings.

Recent innovations in MSC isolation and characterization techniques have further improved the quality and functionality of these cells, allowing for more effective applications in clinical scenarios. Enhanced culture systems, including 3D bioreactors, have been developed to maintain MSC viability and promote osteogenic differentiation, creating a microenvironment that mimics physiological conditions and facilitating the secretion of crucial growth factors and extracellular matrix components essential for bone regeneration (Ling et al., 2024).

Advancements in gene editing technologies, such as CRISPR/Cas9, have enabled the engineering of MSCs to enhance their therapeutic efficacy in bone healing. By modifying MSCs to overexpress specific osteogenic factors or anti-inflammatory cytokines, it is possible to create cell populations with superior regenerative capabilities. Preclinical studies have shown that these genetically modified MSCs can significantly improve bone repair outcomes, reduce inflammation, and enhance the overall healing environment at fracture sites (Maruyama et al., 2021; Li K. et al., 2022).

Clinical trials investigating MSC-based therapies for bone fracture healing are increasingly demonstrating promising results, suggesting that MSC injections can improve healing rates and reduce complications associated with non-union fractures (Arthur and Gronthos, 2020; Re et al., 2023). As our understanding of the mechanisms underlying MSC-mediated bone regeneration increases, the refinement of treatment protocols, including optimal dosing and timing of MSC delivery, is anticipated to enhance the clinical efficacy of these therapies. These advances highlight the importance of MSC-based treatments in improving outcomes in bone fracture healing, providing new hope for patients with complex or delayed healing scenarios.

## 6 Challenges in MSC application for bone fracture healing

### 6.1 Ethical dilemmas

The research and application of MSCs in bone fracture healing present a complex landscape of ethical dilemmas that necessitate careful consideration (Charitos et al., 2021; Harris et al., 2022). One of the primary ethical concerns revolves around the sources of MSCs. Commonly harvested from bone marrow, adipose tissue, and umbilical cord blood, each source brings its own set of ethical implications. Obtaining MSCs from adult donors typically involves invasive procedures that carry health risks. Informed consent is crucial in these scenarios, yet the potential for coercion or undue influence, particularly among vulnerable populations, raises significant ethical questions. On the other hand, while umbilical cord blood is often perceived as ethically acceptable due to its status as discarded tissue, it introduces complications regarding ownership and consent. Parents may not fully grasp the implications of donating cord blood, which can lead to ethical concerns about the adequacy of informed consent processes.

In addition to the sourcing of MSCs, the manipulation of these cells, including genetic modifications, presents further ethical challenges (Feier et al., 2022; de Kanter et al., 2023). Enhancing the therapeutic potential of MSCs through genetic modification can lead to unintended consequences, such as the risk of tumorigenesis or other adverse effects. This necessitates a thorough ethical evaluation of the long-term safety of such modifications, especially given that these cells are intended for therapeutic use in human patients. Moreover, the dual-use nature of MSCs (where techniques developed for therapeutic purposes could also be employed for non-medical enhancements) raises additional ethical concerns. The potential for misuse underscores the importance of establishing guidelines that govern the application of MSC technologies.

Equity and access to MSC-based therapies also warrant ethical scrutiny. As these advanced treatments become integrated into clinical practice, disparities in access may arise, influenced by socioeconomic factors, geographic location, and the availability of healthcare infrastructure. Such disparities could exacerbate existing health inequalities, making it imperative for researchers and healthcare providers to consider their responsibilities in ensuring equitable access to these potentially life-changing therapies. Furthermore, the high costs associated with MSC research and the development of therapies may limit access for some populations, which raises ethical questions about fairness and justice in healthcare.

Effective regulatory oversight is essential to navigate these ethical dilemmas (Harris et al., 2022). Ensuring that clinical trials involving MSCs adhere to ethical standards is crucial for safeguarding participant welfare and maintaining public trust. This includes rigorous review processes that evaluate the risks and benefits of MSC therapies, as well as the ethical treatment of trial participants. Transparency and public engagement play a vital role in fostering trust in MSC research. Engaging diverse stakeholders (including patients, researchers, clinicians, and policymakers) in discussions about the implications of MSC applications can help navigate the complexities of the field and promote ethical governance.

In summary, addressing the ethical dilemmas associated with MSC research for bone fracture healing is essential for the responsible advancement of this promising field. By considering the ethical implications of sourcing, manipulation, equity, and regulatory oversight, researchers and clinicians can work collaboratively to ensure that MSC therapies are developed and implemented in a manner that prioritizes patient welfare, equity, and ethical integrity. This holistic approach may maximize the potential benefits of MSC therapies while minimizing the risks and ethical concerns that accompany their use in regenerative medicine.

### 6.2 Major limitations

As discussed in previous sections, MSCs hold great promises for tissue repair and regeneration due to their ability to differentiate into various cell types, such as osteoblasts, chondrocytes, and adipocytes. Their integration into tissue is facilitated by their capacity to home to injury sites, where they contribute to the repair process through differentiation and secretion of bioactive molecules. However, MSCs have limitations in forming complex structures, as they cannot independently drive morphogenesis or self-organize into intricate, functional tissues. This limitation arises from their inability to establish the spatial organization and signaling required for complex tissue architecture. Therefore, while MSCs are valuable for therapeutic applications in bone and cartilage repair, their inability to form complex structures necessitates strategies that support their organization and differentiation in a controlled manner. Such strategies might include using scaffolds, growth factors, or co-culturing with other cell types to guide the formation of more complex tissue structures. Understanding these aspects is of primary relevance for advancing MSC-based therapies and ensuring successful tissue regeneration outcomes.

Another significant challenge in the application of MSCs is their inability to effectively migrate to the fracture site following delivery. When administered systemically, a substantial proportion of MSCs may become trapped in organs such as the lungs and liver, rather than reaching the targeted tissue necessary for healing. This misdirection can lead to suboptimal therapeutic outcomes, as the MSCs may not efficiently localize the injury site to exert their regenerative effects (Najar et al., 2022). To overcome this challenge, strategies such as enhancing MSC homing through genetic modification, using targeted delivery systems, or employing chemotactic agents can be explored to improve their localization and effectiveness at the injury site.

In addition, the biological variability of MSCs presents a significant challenge in their therapeutic application. This variability is influenced by factors such as the source of MSCs, donor characteristics, and the conditions under which they are cultured, which can lead to varying therapeutic outcomes. For example, MSCs from different sources share common features but also display distinct gene expression profiles and functional properties (Lyu et al., 2024). Donor-specific factors such as age, sex, body mass index, and underlying health conditions further influence MSC phenotype, morphology, differentiation potential, and function, potentially impacting treatment effectiveness (da Silva et al., 2025). This variability makes it challenging to compare outcomes across different clinical trials and to establish universally effective treatment protocols, as inter- and intra-donor heterogeneity can significantly impact results (Calcat-I-Cervera et al., 2023). Additionally, donor-to-donor and intra-donor variability introduces another layer of complexity, as MSCs from different individuals may respond differently to the same therapeutic intervention, further challenging reproducibility and consistency in their effects (Trivedi et al., 2024).

Furthermore, variations in the preparation of MSC products introduce additional heterogeneity. Differences in cell culture media composition, the presence or absence of growth factors, and culturing techniques can significantly impact the efficacy of MSC-based therapies (Česnik and Švajger, 2024). The storage protocols, including cell concentration and delivery solutions, are also essential for maintaining cell viability (Maličev and Jazbec, 2024). In addition, discrepancies in administration protocols can critically affect the distribution and functionality of administered cells, leading to inconsistent clinical results (Li et al., 2023). This variability highlights the need for standardized practices to enhance the reliability and efficacy of MSC-based therapies (Wright et al., 2021).

To address these challenges, strategies such as standardizing culture conditions, selecting MSCs based on specific markers associated with enhanced regenerative potential, and employing bioreactor systems to optimize cell growth and differentiation can be implemented. Pooling MSCs to create more homogeneous populations and harmonizing assessment methods for their specific functions are also crucial steps towards reducing product heterogeneity. These approaches can help ensure more consistent outcomes in MSC-based therapies for bone regeneration, as summarized in Table 2.

**TABLE 2:** Summary of factors contributing to MSC variability and their impact on therapeutic efficacy. This table highlights the diverse sources and conditions affecting MSC characteristics and suggests potential strategies for achieving more consistent outcomes in MSC-based therapies.

Factor	Source	Impact on MSC variability
Tissue Source	Bone marrow, umbilical cord, adipose	Different gene expression profiles and functional properties
Donor Characteristics	Age, sex, BMI, health conditions	Influences phenotype, morphology, differentiation potential, and function
Culture Conditions	Media composition, growth factors	Variability in MSC preparation affecting efficacy
Storage Protocols	Cell concentration, delivery solution	Affects cell viability and post-thawing protocols
Administration Protocols	Delivery method	Impacts distribution and functionality of MSCs

Another limitation in the therapeutic application of MSCs is their general scarcity (Fernández-Santos et al., 2022). This limited availability necessitates *ex vivo* expansion to obtain a sufficient number of cells for clinical use. However, the process of *ex vivo* expansion is often time-consuming, requiring several weeks to generate a clinically relevant cell population. During this expansion phase, there is a risk of compromising the functionality of the MSCs. Prolonged culture periods can lead to alterations in the cells’ biological properties, including their proliferation rates and differentiation potential. These changes may decrease their ability to effectively contribute to tissue regeneration upon transplantation (Nikolits et al., 2021). Also, the expansion process can induce cellular stress responses, which may increase the likelihood of senescence (a state of irreversible growth arrest that negatively impacts the regenerative capacity of MSCs) (Olmedo-Moreno et al., 2022). Moreover, the risk of cellular transformation during expansion raises concerns about the long-term safety of MSC therapies (Costa et al., 2021). Such transformations could potentially lead to tumorigenesis, posing significant risks to patients receiving MSC-based treatments. Therefore, while *ex vivo* expansion is essential for obtaining an adequate number of MSCs, it introduces several challenges that must be carefully managed to ensure the efficacy and safety of MSC therapies in clinical applications. To address these limitations, strategies such as optimizing culture conditions to minimize stress, employing small-molecule compounds to enhance MSC survival and function during expansion, and using bioreactors for more controlled and efficient cell growth could be implemented. These approaches are crucial for optimizing the use of MSCs in regenerative medicine and improving patient outcomes.

## 7 Future perspectives and innovations in MSC therapy

### 7.1 Emerging technologies in MSC engineering and delivery

#### 7.1.1 3D bioprinting with MSCs

MSCs are a prevalent cell type in the contexts of 3D bioprinting, where they are known as bioink. MSCs are not used only for bone, cartilage or adipose tissues but were also deeply involved in several other applications of 3D bioprinting (Kara Özenler et al., 2024). This section presents an understanding of MSCs physiology important in development of efficient 3D bioprinting solutions to produce dependable MSC products. MSCs have complex physiological features and depend on the micro surrounding area in which they are found. Some authors have identified the tumor-suppressive properties of MSCs (Sun et al., 2022). However, MSCs can also contribute to tumor progression by raising blood vessel formation inside the tumor, strengthening the formation of the tumor, and by liberating several biologically active factors (Liang et al., 2021; Li K. et al., 2022; Zhao et al., 2021).

Bioprinting has been studies for bones tissue fabrication due to its potential of fabricating patient -specific tissue constructs that replicate anatomical structures (Wang M. et al., 2025). In a pioneer study, Gao et al. used a simple thermal inkjet bioprinter to fabricate PEGDMA scaffolds (Gao et al., 2014). Human mesenchymal stem cells (hMSCs) derived from bone marrow were printed together with NPs of bioactive glass and HA during the process of polymerization of the solution. The study also showed that using bioprinting, there was uniform distribution of hMSCs within the scaffold, while, if hMSCs were piled onto the scaffold manually, hMSC aggregated at the lowest part of the scaffold because of gravitational forces. The successful generation of brain tissues using MSCs was already also described (Restan Perez et al., 2021; Layrolle et al., 2022); the bioink used here was fibrin. This method enabled the creation of semi-spherical structures in shape which promoted the change of MSCs into cells similar to neurons. When the structures were stored on the bioprinter platform, cell vitality remained intact and there was improved cell organization over time, indicating they could be used to model neural disorders and assess treatments for neurological conditions. Furthermore, the development of agarose bioinks has shown promise for 3D bioprinting in bone tissue engineering, with modifications allowing for improved printability and mechanical properties, which support the osteogenic differentiation of MSCs (Augustine et al., 2024).

In addition, the application of bioactive materials has been highlighted as a critical strategy for enhancing osteogenic function in bone tissue engineering, with various bioactive components promoting cellular adhesion and proliferation (Bai et al., 2024). Calcium phosphate biomaterials have emerged as essential components in bioinks, providing the necessary mechanical and biological properties for effective bone regeneration (Tolmacheva et al., 2024). The versatility of 3D printing technology allows for the precise fabrication of scaffolds that can mimic the complex architecture of native bone, thereby improving patient outcomes and reducing the risk of rejection (Jalise et al., 2025). This integration of advanced biomaterials and printing techniques positions bioprinting at the forefront of innovative solutions for addressing bone defects and advancing regenerative medicine.

#### 7.1.2 Nano-carrier system in MSC therapy

Silica nanoparticles and polymeric nanoparticles are designed to encapsulate growth factors or treatment agents that promote bone healing. These carriers have the potential to enhance dispersibility and stability of osteogenic drugs to allow targeted delivery at the fracture site (Güven, 2021; Prasad et al., 2021). Nanoparticles may transport BMPs or other agents that can encourage bone regeneration and therefore improve healing (Wen et al., 2023). This is accomplished by making sure that therapeutic concentrations of such agents are present at the site of injury for a long period.

Biologically inspired hydrogels that can nucleate and accommodate MSCs and therapeutic lend a structure to the bone regeneration process (Bai X. et al., 2023; Sun et al., 2023). These biomaterials have the purpose of reproducing the function of the EM and to stimulate the adhesion of MSCs, as well as to control the release of the growth factors. Hydrogels that have this conjugation for the purpose of combining with MSCs and osteogenic factors demonstrate improved bone healing outcomes in preclinical studies (Xu et al., 2024). This is realized through the successful induction of MSCs into osteoblasts before enhancing bone formation. The delivery system may comprise biodegradable polymer microspheres that can encapsulate MSCs and deliver them, along with growth factors, to the area of the fracture (et al., 2024). The progressive delivery system significantly enhances the gradational distribution of osteogenic factors within the immediate affected area. This targeted approach ensures that the site of injury receives a concentrated dose of these critical factors, while the rest of the body is exposed to a much smaller amount. As a result, the method optimizes the healing environment at the fracture site and minimizes potential side effects associated with higher systemic doses, promoting a more effective and safer therapeutic outcome.

Research experiments revealed that these systems can significantly enhance the activity of bone formation and healing in requests of large bone defects. Nanocomposite systems blend nanoparticles with standard biomaterials to build frameworks that support the growth and differentiation of MSCs (Andalib et al., 2021; Hung et al., 2024). It is possible to design these systems to have specific mechanical properties and rates of degradation which would correspond to the time it takes to heal a break in a patient’s bones. Calcium phosphate cements have been mixed with nanoparticles to improve the ability of scaffolds to support bone growth and increase their strength (Sutthavas et al., 2022). Additionally, this combination allows for a controlled and continuous release of medicinal substances.

Another area that has gained significant attention for MSC therapy in bone regeneration is the use of lipid nanoparticles (LNPs). Lipid nanoparticles provide remarkable biocompatibility, a controlled drug release mechanism, and the ability to target specific tissues passively, making them ideal candidates for delivering therapeutic agents to bone lesions (Xu et al., 2023). However, challenges such as difficulties in transport, storage, and maintaining drug concentration at the target site limit their clinical application (Burduşel and Andronescu, 2022). Recent studies have highlighted the efficacy of LNPs in facilitating the osteogenic differentiation of MSCs, particularly through the silencing of suppressor genes like GNAS, which has shown promise in enhancing bone formation in animal models (Basha et al., 2022). This approach not only improves the differentiation of MSCs into osteoblasts but also provides a novel strategy for treating conditions like osteoporosis, where traditional therapies fail to stimulate new bone formation (Basha et al., 2022). Furthermore, advancements in the design of ionizable lipids have led to the development of more effective LNP formulations, which have been shown to enhance mRNA delivery for osteogenic factors, such as β-catenin, thereby promoting bone healing and regeneration (Nelson et al., 2024). The integration of these innovative lipid-based systems with existing scaffold technologies could potentially overcome the limitations of traditional scaffolds, which often lack sufficient bioactivity to effectively support tissue regeneration (Hallan et al., 2022). Overall, the combination of LNPs with MSC therapy represents a promising Frontier in bone regeneration research, leading to new therapeutic strategies that could significantly impact the treatment of bone-related diseases.

### 7.2 Potential role of MSCs in aging and osteoporosis-related fracture

The processes causing the delayed fracture healing in older people compared to younger patients are not completely known (Duda et al., 2023). Several age-related changes have been suggested, including modified interaction between macrophages and MSCs (Duda et al., 2023). Multiple networks of connections interact to operate the aging mechanism of MSCs. The primary indicators of aging in MSCs are genetic material damage, noncoding RNA and exosomes, loss of proteostasis, intracellular signaling pathways, and mitochondrial malfunction (Fraile et al., 2022). Aging MSCs present a decline in their ability to repair DNA and their capacity to counteract oxidative stress, making them more prone to the development of tumors and DNA damage (Al-Azab et al., 2022). New data suggest that MSCs derived from the later stages of human bone marrow-derived MSCs exhibit pronounced signs of aging, including alterations in their immune characteristics and physical morphology (Rasouli et al., 2024). In contrast, MSC aging is linked to increased oxidative stress, heightened oxygen consumption, and genomic instability (Denu and Hematti, 2021; Buzoglu et al., 2023). Given these factors, it is advisable to assess the proportion of aneuploid cells within MSC populations prior to their application in clinical settings. This evaluation is critical for selecting the appropriate strategies to mitigate MSC senescence and optimize their therapeutic potential. By addressing these age-related changes, we can enhance the efficacy of MSCs in regenerative medicine.

The application of MSCs and their derived osteoprogenitors in orthopedic surgery is increasingly gaining recognition. Pioneering studies by Hernigou et al. (2016) established the use of osteoprogenitors derived from MSCs for the treatment of osteonecrosis of the hip and other bone healing-related conditions. Recent studies have further demonstrated the feasibility of using harvested osteoprogenitors for addressing secondary osteonecrosis of the knee (Boontanapibul et al., 2021). As MSCs undergo dynamic alterations throughout the aging process, their self-renewal capacity, differentiation potential, and migratory abilities significantly decline. These changes are often associated with a senescent phenotype, which results in a permanent state of growth arrest and resistance to apoptosis. Both activated and senescent MSCs contribute to inflammation, leading to a phenomenon known as “inflammaging,” which exacerbates age-related conditions such as osteoporosis. Furthermore, senescent MSCs secrete cytokines through the senescence-associated secretory phenotype, impairing the function of neighboring MSCs and other cells involved in bone regeneration. The use of autologous MSCs in elderly patients presents challenges due to the adverse microenvironment that negatively impacts MSC function, resulting in fewer positive outcomes compared to younger cohorts.

To enhance the reparative potential of aged MSCs in fracture healing, various strategies are being explored. These include optimizing culture conditions to maintain MSC viability and functionality, employing senolytic agents to selectively eliminate senescent MSCs, and using genetic manipulation techniques to bolster the regenerative capabilities of stem cells (Menche and Farin, 2021; Chen Y. Q. et al., 2022). Focusing on rejuvenating the aging microenvironment may significantly enhance the efficacy of aged MSCs. The application of youthful serum or specialized growth factors has been shown to improve the functionality and performance of aged MSCs. Furthermore, combining MSC therapy with pharmacological treatments designed to stimulate new bone tissue formation demonstrates considerable potential in improving healing outcomes for fractures, especially in patients with osteoporosis (Shim et al., 2021; Qian et al., 2024). These synergistic approaches aim to augment the regenerative capacity of MSCs, thereby enhancing the overall healing process in older populations. By addressing the challenges associated with aging, these strategies hold promises for optimizing therapeutic interventions in regenerative medicine.

## 8 Conclusion

Understanding the functional roles of MSCs in osteogenesis contributes to advancements in bone fracture healing. This review presents a comprehensive overview of significant developments in MSC research encompassing cell derivation, manipulation, and application. Different methodologies for bone regeneration demonstrate promise for addressing extensive bone defects, whether in normal or infected conditions. The primary function of transplanted MSCs extends beyond merely increasing the population of existing MSCs to restore bone healing capacity; they also exert considerable influence through the molecular components they secrete, which regulate and modulate both their own behavior and the surrounding microenvironment, including in the context of skeletal diseases. As research and development progresses, MSC-based therapies are available to transform orthopedic medicine, potentially leading to improved outcomes for patients with bone-related complications. However, several preclinical and clinical translation challenges must be addressed prior to and during MSC-mediated bone healing, particularly concerning bone fractures. Addressing these limitations is essential for realizing the full therapeutic potential of MSCs in clinical settings.
